# An assessment of the Zimbabwe ministry of health and child welfare provider initiated HIV testing and counselling programme

**DOI:** 10.1186/1472-6963-12-131

**Published:** 2012-05-28

**Authors:** Euphemia L Sibanda, Karin Hatzold, Owen Mugurungi, Getrude Ncube, Beatrice Dupwa, Pester Siraha, Lydia K Madyira, Alexio Mangwiro, Gaurav Bhattacharya, Frances M Cowan

**Affiliations:** 1 ZAPP-UZ, Department of Community Medicine, University of Zimbabwe College of Health Sciences, Harare, Zimbabwe; 2 Centre for Sexual Health and HIV Research, University College London, London, UK; 3 Population Services International, Harare, Zimbabwe; 4 Zimbabwe Ministry of Health and Child Welfare, Harare, Zimbabwe; 5 Clinton Health Access Initiative, Harare, Zimbabwe

## Abstract

**Background:**

Provider-initiated HIV testing and counselling (PITC) is widely recommended to ensure timely treatment of HIV. The Zimbabwe Ministry of Health introduced PITC in 2007. We aimed to evaluate institutional capacity to implement PITC and investigate patient and health care worker (HCW) perceptions of the PITC programme.

**Methods:**

Purposive selection of health care institutions was conducted among those providing PITC. Study procedures included 1) assessment of implementation procedures and institutional capacity using a semi-structured questionnaire; 2) in-depth interviews with patients who had been offered HIV testing to explore perceptions of PITC, 3) Focus group discussions with HCW to explore views on PITC. Qualitative data was analysed according to Framework Analysis.

**Results:**

Sixteen health care institutions were selected (two central, two provincial, six district hospitals; and six primary care clinics). All institutions at least offered PITC in part. The main challenges which prevented optimum implementation were shortages of staff trained in PITC, HIV rapid testing and counselling; shortages of appropriate counselling space, and, at the time of assessment, shortages of HIV test kits. Both health care workers and patients embraced PITC because they had noticed that it had saved lives through early detection and treatment of HIV. Although health care workers reported an increase in workload as a result of PITC, they felt this was offset by the reduced number of HIV-related admissions and satisfaction of working with healthier clients.

**Conclusion:**

PITC has been embraced by patients and health care workers as a life-saving intervention. There is need to address shortages in material, human and structural resources to ensure optimum implementation.

## Background

In countries with generalised HIV epidemics HIV testing is the gateway to HIV treatment, care and support of HIV infected and affected individuals. In 2007 WHO issued guidelines for Provider-Initiated HIV Testing and Counselling (PITC), recommending that for countries with generalised HIV epidemics, testing should be offered to all individuals presenting at health care institutions [1].

The introduction of PITC is associated with positive outcomes: in South Africa it increased the proportion of new patients presenting at sexually transmitted infection clinics who were tested for HIV [2] and increased the proportion of tuberculosis patients who received HIV counselling and testing [3]. PITC has increased the number of children tested for HIV and commenced on antiretroviral therapy (ART) in Malawi [4] and is associated with an increase in knowledge of partner HIV status in Uganda [5]. There is also evidence that PITC is feasible and acceptable to patients and caregivers [6-9][10].

Although research among health care workers internationally/regionally suggests that they are positive about the benefits of PITC, there is also evidence that in resource-constrained settings PITC scale-up is hampered by shortages of staff, confidential counselling space and clinic supplies [11-14]. This may affect the quality of services provided. Task-shifting to lay counsellors has been demonstrated to be an effective strategy that helps nurses deal with the increased work load that PITC inevitably brings [15-17]. There has also been concern that because of the power imbalance between patients and health care workers, patients may feel less able to refuse HIV testing when health care workers recommend it [12]. However the high refusal rate in a PITC trial in South Africa shows that patients were able to exercise their right to refuse testing [2].

The Zimbabwe Ministry of Health and Child Welfare (MoHCW) introduced PITC in 2007 stating that anyone presenting to any level of health care institution should be offered HIV testing regardless of the purpose of their visit [18]. Here we present results of the first formal evaluation of this programme, which was aimed to evaluate institutional capacity to implement PITC and to investigate patient and health care worker (HCW) perceptions of the programme. Such an evaluation is important as it enables formulation of evidence-based strategies for optimum implementation of PITC in Zimbabwe and other low income countries with high HIV prevalence.

## Methods

### Selection of study sites

By the time of this study, all health care centres in Zimbabwe (about 1,600 in total) were supposed to be implementing PITC. However MoHCW was aware through monthly reports provided by sites that many sites were not implementing this service. Purposive sampling of study sites was conducted to include health care institutions which were actively implementing PITC as evidenced by submission of regular reports to MoHCW. Sites were selected across provinces to ensure representation of all levels of care (i.e. from rural and urban primary health clinics to central hospitals).

### Data collection

At each participating institution, study procedures were conducted during one working day. Procedures were divided into three:

1. **Assessment of implementation activities and institutional capacity to provide PITC**A semi-structured interview was conducted in English with the head/designee of each facility. They were asked about PITC implementation activities and adequacy of human, material and structural resources for implementation.

2. **In-depth interviews with patients/attendees**Convenience sampling of patients/attendees was conducted: patients/attendees who had been offered HIV testing at the participating centres on the day of the study visit, regardless of whether they accepted, were invited for in-depth interviewing. They were asked about awareness of PITC and perceptions of the programme. Written informed consent was obtained from participants before interviewing. Interviews were conducted by four trained field workers with a medical background who had experience in conducting qualitative interviews in various communities in Zimbabwe. Interviews were conducted in the language of the participant (Shona or Ndebele), transcribed verbatim and then translated into English. Interviews typically lasted about forty-five minutes.

3. **Focus Group Discussions (FGDs) with Health Care Workers (HCW)**The head/designee of each institution was asked to refer HCW involved in PITC implementation to study staff. HCWs were invited to take part in FGDs and those who agreed were asked to provide written informed consent. The groups comprised 6–8 participants but in smaller sites discussions were held with the few available nurses and primary counsellors. Discussions were in English and focused on HCW experiences of PITC implementation and their attitude towards the programme.

### Qualitative data collection and analysis

In-depth interviews and FGDs were digitally-recorded, transcribed and translated into English. To ensure the accuracy of transcriptions and translation, 20% of transcripts were randomly selected and checked by two of the field workers. Data were entered into NVIVO 8 (qualitative data management software). Data analysis was done according to principles of Framework Analysis [19]. Familiarisation with the data began during the data collection process through listening to tape recordings, reading transcripts and interview notes. From the study objectives and issues raised by participants we identified the coding framework which was used to systematically code the textual data. The data were subsequently arranged into themes and subthemes and illustrated with verbatim quotes.

### Ethical approval

This study was approved by the Medical Research Council of Zimbabwe.

## Results

The study was conducted in two central, two provincial and six district (two urban, one peri-urban and three rural) hospitals and six primary care clinics (five urban, one rural) in six of Zimbabwe’s ten provinces.

### Assessment of implementation activities and institutional capacity to provide PITC

This assessment was conducted in full in all but one clinic; at that clinic it was partially completed because the site leader was unavailable to complete the assessment. Table 1 provides a summary of implementation activities and institutional capacity to provide PITC.

**Table 1:** Site capacity to implement HIV Testing and Counseling (HTC) services

**Domain**	**Number in Central Hosp (2)**	**Number in Provincial Hosp (2)**	**Number in District Hosp (6)**	**Number in Clinic (6)**	**Total (16)**
**Staff skills/responsibilities**					
Availability of job descriptions for staff delivering HTC services					
Yes	1	2	3	2	8
No	1	0	2	4	7
Availability of standard operating procedures for upholding confidentiality and informed consent					
Yes	2	2	5	2	11
No	0	0	1	4	5
Interviewer’s opinion on commitment and level of interest of leadership in HTC programme					
Highly committed	2	2	5	6	15
Moderately Committed	0	0	1	0	1
Not committed	0	0	0	0	0
**Adequacy of facilities**					
Adequate space for confidential counselling					
Yes	2	0	0	1	3
No	0	2	6	4	12
Adequate of space for confidential HIV testing					
Yes	2	1	4	3	10
No	0	1	2	2	5
Availability of secure storage facilities					
Yes	2	2	5	3	12
No	0	0	1	3	4
**Implementation activities**					
Number of days per week HTC services available					
5 days	1	1	2	2	6
7 days	1	1	4	3	9
Provision of outreach activities					
Yes	0	1	4	0	5
No	2	1	1	6	10
Availability of male condoms					
At time of visit	2	2	6	5	15
Always available	2	2	6	4	14
Not always available	0	0		1	1
Availability of female condoms					
At time of visit	2	2	6	4	14
Always available	1	2	3	4	10
Not always available	1	0	2	1	3
Provision of cotrimoxazole prophylaxis					
Yes	2	2	6	5	15
No	0	0	0	0	0
Provision of fluconazole prophylaxis					
Yes	2	2	6	1	11
No	0	0	0	4	4
Provision of ART					
Yes	2	2	6	5	15
No	0	0	0	1	1
Provision of PITC for TB patients					
Yes	2	2	6	4	14
No	0	0	0	1	1
TB screening for HIV positive patients					
Yes	2	1	4	3	10
No	0	1	2	2	5
Provision of TB treatment					
Yes	2	2	6	4	14
No	0	0	0	1	1
Availability of PEP guidelines					
Yes	2	2	6	4	14
No	0	0	0	1	1
**Primary Counsellor Evaluation**					
Availability of Primary Counsellors					
Yes	2	2	6	5	15
No	0	0	0	1	1
Who pays Primary Counsellor monthly allowances					
Global Fund	1	1	5		7
Expanded Support Program (ESP)			1	1	2
Government	1	1			2
Local Authority				2	2
**Support and Supervision from higher level**					
Did staff from the higher levels visit the site to supervise data collection and management during the past three months?					
Yes	0	2	6	2	10
No	1	0	0	3	4

HTC=HIV testing and counselling services.

ART=Antiretroviral drug therapy.

PEP=Post-Exposure Prophylaxis.

#### Staff Skills and responsibilities

All sites had staff (nurses and laboratory technologists in hospitals and nurses only for clinics) who were trained in rapid HIV testing, although in most instances there were too few. Most testing was done by nurses, with laboratory technologists providing quality control. Where HIV testing was conducted by laboratory technologists, the fact that samples were tested in the laboratory caused a delay in patients receiving their results, resulting in some patients leaving the facility without results.

Most (15) sites had the Primary Counsellor (PC) cadre. This is a newly created non-clinical cadre who are trained to provide HIV-related counselling. PC duties included conducting group HIV education sessions, pre and post-test counselling, and antiretroviral therapy (ART) adherence counselling.

#### Adequacy of facilities

In ten sites there was inadequate space to provide confidential counselling. In contrast, most sites (n = 12) had adequate facilities to assure confidential HIV testing. Only central hospitals, which are much larger, did not report problems with space.

#### Stock levels

During the time of assessment most health care institutions had inadequate stocks of HIV test kits and so were unable to offer PITC services to all patients/clients.

None of the institutions reported problems with antiretroviral drug stocks.

#### Implementation activities

Only one site implemented PITC according to MoHCW guidelines. In all other sites PITC was implemented partially because there were shortages of HIV test kits and inadequate numbers of staff trained in HIV testing and counselling or PITC. This resulted in two main deviations 1) testing was initiated only for priority patients/attendees, e.g. those who were suspected to have HIV-related illness and for PMTCT purposes; 2) Instead of offering HIV testing before consultation (as per MoHCW guidelines) it was offered after patients had completed the consultation process.

### Patient views on PITC

36 patients/clients were interviewed, 31 of whom were female (Table 2). Participant ages ranged from 18–73 years.

**Table 2:** Characteristics of Interviewed Patients/Clients

Characteristic	N
Sex	
Male	5
Female	31
Age (years)	
20-	4
21-25	10
26-30	8
31-35	6
36-40	1
41-45	3
46-50	0
>50	4
Reason for Clinic Visit	
Primary Care	12
Antenatal Care	13
In-patient	2
VCT	4
Other	5
Facility attended	
Central Hospital	4
Provincial Hospital	4
District Hospital	18
Clinic	10
HIV status	
Positive	12
Negative	20

#### Awareness of the existence of PITC

Most patients reported that they were aware of the existence of HIV testing and counselling (HTC) services at health care institutions, through friends/relatives in the community, or via the media or through HCW. However some were unaware that the testing was provider-initiated. Many of those interviewed reported that HTC services were not always available.

"“They said you should come (for HIV testing) on Mondays, Wednesdays and Fridays…” (42-year old woman whose purpose of visit was VCT)."

A few patients (n = 4, two of whom came from rural areas) were not aware of the existence of HTC services at health care institutions.

#### Patient/Client Perceptions of PITC

PITC was viewed positively by all participants, including the two who had opted out of testing. Most said it has enabled people living with HIV to live normal lives following early diagnosis and timely initiation of treatment.

"“It (PITC) is good because if you have HIV you know that you will then take tablets and live a longer life. If you have a family then you will take care of them.” (27 year old woman whose purpose of visit was VCT)"

Many patients viewed PITC as a more effective means of getting people tested than VCT believing that fear makes it difficult for people to volunteer to get tested.

"“…One will never be able to volunteer unless someone tells them to get tested; they are scared that maybe they will be found to be infected.” (23 year old woman who had come for antenatal care)"

Some participants confessed that they too had been fearful before they received support from health care workers.

"“I am one of those who said we will see to it (HIV testing) when we fall sick…I actually told myself I wouldn’t be able to handle it…I would commit suicide.” (33-year old woman who had come for ANC)"

Furthermore, PITC has provided an opportunity for people who previously did not know where/how they could access HIV testing.

"“Since last year I have wanted to get tested but didn’t know where to go…” (56 year old woman who was seeking primary care)"

#### Quality of service provided

Most patients reported that the education they received from health care workers was sufficient to enable them to make decisions about testing. The quality of counselling and treatment services provided by health care workers was highly regarded.

*“…they properly teach us so that we understand. They don’t get cross with us, no, they talk to us nicely; we are free and they are also free.”* (42 year old woman who had come for VCT)

However, a few patients reported that they had not received adequate information before testing: they said they were simply referred for testing. In addition, although it was clear to most patients that their consent was required before testing, some did not feel able to opt-out because they felt it would frustrate the HCWs who needed the HIV result to plan their treatment. This is illustrated by this quote from a 45 year old woman who had just tested HIV positive and was explaining why she felt compelled to get tested.

"“I thought that (if I refused) they would say so how do you want us to treat you.”"

Furthermore, some patients perceived that the nurses were too busy to provide quality counselling.

Some patients reported that they were confused by the client flow and did not know where to go after group education, or HIV testing.

"“… we didn’t know where to go if you want to get tested. We kept asking (for directions).” (27-year old woman who had come for VCT)"

Some patients who had received group education/offer of HIV test said they did not feel able to ask questions if they did not understand. They were worried about what other patients would think or that other patients would disclose what had been said during the group education session to people in the community.

"“They will go about saying that woman said this and that.”(34-year old woman who had come for ANC)"

#### Participant’s awareness of post-test support services

While most patients were aware of the availability of post-test medical/clinical services such as antiretroviral therapy and PMTCT services, there was limited awareness of psychosocial support services that are available to HIV infected individuals.

#### Feelings about testing decision

None of the patients regretted their decision to get tested. Those who tested negative were naturally pleased about their result and their decision to get tested. Some patients who had tested positive were relieved to know the cause of their symptoms and felt they would now receive appropriate care/treatment.

"“I am happy that although I was found to have the disease I know that I will get a lot of help.” (29-year old woman who was seeking primary care)"

"Right now I feel happy because when you have been told you have it (HIV) you now have the knowledge that if I fall sick I will go to the hospital where I will get help. If I had been sitting at home and I got sick I might have wasted time visiting (traditional healers) a n’anga, or a prophet… (30 year old woman who had come for VCT)."

### Health Care Worker (HCW) views on PITC

#### HCW views of what had worked in the PITC programme

##### i) Reduction in illness and death

In all institutions HCW agreed that scaling up HTC services and provision of ART had resulted in patients living with HIV being healthier due to earlier detection and timely access to treatment. The number of patients arriving at health care centres moribund had apparently decreased and there were fewer admissions for treatment of opportunistic infections than previously. This represented a huge motivation for HCW.

"“I am happy because they (patients) are coming while they are still able to move unaided.”(Female registered nurse (midwife), primary care clinic)"

"“Yes, HIV testing is also good because I’ve noticed that the number of people who are being admitted in the wards, it’s now different from long ago like 2005, 2006 where there was no ART and cotrimoxazole. Bed-ridden patients are very few now… .”(Female nurse, District Hospital)"

"“When there are less deaths the job is more interesting.” (Female registered nurse, District Hospital)"

##### ii) Increased patient confidence in health care system

In the past sick HIV positive patients often had to visit health care institutions repeatedly with unresolved illness. With the introduction of PITC HIV positive patients had a higher chance of getting appropriate care. Nurses reported that this had increased patient confidence in the health care system.

"“And the other benefit I realise with PITC is the patients have more confidence in the health system. Like the patient will come today with a cough, you treat them. Suppose there was no PITC? They’ll come today with a cough. You treat them. They go. They come the next time with an abscess…” (Female registered nurse, primary care clinic)"

##### iii) Reduced stigma

In the past when a patient was offered HIV testing by a HCW they would think the provider had noticed a sign/symptom that may be attributable to HIV and they would feel anxious. PITC has resulted in an atmosphere of open discussion about HIV in health care settings. This openness has spread to communities where patients feel comfortable to ask questions about HIV infection or care even in public settings. Many providers said patients would now stop them in the street to ask questions about HIV and HIV-related care and treatment.

"“…They will come, even when there are 15 or so people around and ask you, “XX, I want to come and collect my cotrimoxazole. Is it now available?”” (Male nurse, primary care clinic)"

Most HCWs reported a huge increase in uptake of HIV testing as a result of PITC. As the number tested increased, the proportion testing negative also increased. Health care workers found this very motivating.

##### iv) Reduction in mother-to-child transmission of HIV

Since the scale up of PITC, nurses perceive that fewer babies born to HIV positive mothers become infected with HIV.

"… “I’m finding that among those mothers who went on this programme and they were Code 1 (HIV positive), all their babies are negative!” (Female registered nurse, central hospital)."

#### *Challenges in scaling up PITC*

##### i) Inadequate space

The scale up of PITC has resulted in increased demand for space for confidential counselling. Many health care institutions were built before the HIV epidemic. Health Care Workers observed that available rooms often failed to provide adequate privacy and confidentiality while others were inadequately ventilated.

##### ii) Shortages of trained staff

Shortages of staff trained in a) implementation of PITC, b) rapid HIV testing and c) counselling was reported to hinder the smooth implementation of PITC.

In most institutions few staff had been selected to undergo PITC training in workshops which were held away from the health care institutions. There was perceived financial benefit from workshop attendance (from payment of travel and subsistence allowances), which has created negative attitudes among staff who were not selected to attend the training workshops. Those who had attended the workshops felt they did not get support from their counterparts who had not been trained in workshops.

"“They say you have had some whatever, HIV/AIDS… what, what workshops and the like and we haven’t attended. So since you have attended, just go and do it yourself” (Female nurse, district hospital)"

"“They also argue that you are paid during your training. You got the T&S (the travel and subsistence allowances), you were paid for it so do it yourself.”(Male nurse, another district hospital)"

##### iii) Shortages of HIV test kits

At the time of the assessment many health care institutions had had stocks-outs of HIV rapid test kits (November 2009 to January 2010). As a result HIV testing and counselling services were either not offered at all or were given to priority patients.

##### iv) Availability of CD-4 count machines

Many institutions did not have enough capacity to provide CD4 count testing for patients who tested HIV positive due to a) unavailability of CD4 count testing devices, b) frequent break-down of CD4 testing devices and c) shortage of required reagents to conduct CD4 cell count. This caused delays in initiation of ART for many patients and was a source of frustration for health care workers.

"“…you also become frustrated as the nurse because you have encouraged this patient to test and this patient now wants a CD4 count…but they can’t access it…” (Male registered nurse, primary care clinic)"

### Effect of PITC on workload

In all institutions health care workers stated that scaling up HTC services had increased their workload, particularly for the opportunistic infections and ART clinics. In most (15) sites this was not viewed negatively; health care workers were motivated by working with a healthier clientele. They acknowledged that although the workload in the HIV clinics had increased, there had been a reduction in workload in other outpatient departments and in-patient wards.

"“Surely the workload has increased, but on the other hand, it has dramatically been reduced. They take their drugs and they improve. Their health improves. So, the time they spend at the hospital, it’s now limited….” (Male nurse, District Hospital)"

## Discussion

This assessment revealed that all assessed health care centres were implementing PITC at least partly. Shortages of human, material and structural resources prevented the optimum scale-up of the programme. Both patients and HCWs have embraced PITC as a life-saving intervention.

There were inadequate facilities for confidential HIV counselling in 12 of 15 sites. This is critical for the success of HIV testing programs as perceived lack of confidentiality may be a barrier to testing [20-22].

Also of note was the stock-out of HIV test kits in many health care institutions at the time of assessment. These stock-outs were caused by increased demand for HIV testing outstripping the supply of test kits. There is need to improve the forecasting of all commodities required for PITC to ensure the success of the programme (there are anecdotal reports that the stocks have since improved as a result of better forecasting).

In all institutions there were shortages of trained staff. Also of note was the discord between staff who had attended training workshops and those who had not. In addition half of the sites did not have job descriptions for staff involved in HIV testing services, which may have affected implementation as some staff were not aware of their specific PITC role. Staffing levels have not been increased to accommodate scale-up of PITC and indeed many institutions have staff vacancies as a result of outmigration of HCWs, resulting in extreme pressure on the remaining staff. It is obviously important to train more nurses. PITC training should be offered to all staff who are expected to provide it to avoid the discord that was reported by the health care workers. In addition, task shifting to the newly introduced primary counsellor cadre across all sites will ease workloads. In this assessment nurses reported that the primary counsellors (whose responsibility was to provide HIV-related counselling) enabled nurses to cope with the extra workload introduced by PITC. To reduce the nurses workload further the Zimbabwe Ministry of Health has now trained the primary counsellors to also conduct rapid HIV testing. This will improve the quality of care as nurses can focus on clinical tasks. It will do away with the reported discomfort patients felt when they were being counselled and tested by a nurse who appeared very busy or clearly needed to attend to more seriously ill patients. Task-shifting has been reported as being effective in other settings [16,23-25].

It is encouraging to note that despite the shortages in resources there is evidence that coverage of PITC and ART services continues to increase with the number of people testing for HIV increasing from 750,000 in 2009 to 1,000,000 in 2010 [26] and the number of patients receiving ART in Zimbabwe increasing from 150,000 in January 2009 [27] to 315,000 in December 2010 [28].

Patients have embraced PITC which is perceived to have resulted in healthier people living with HIV through timely access to appropriate treatment. The acceptability of PITC among patients has been reported in other countries in Sub-Saharan Africa [6,8,10,29]. The negative aspects that patients reported about the programme (eg incomplete information giving during counselling) can be solved by resolving challenges with human resources.

Although HCW hugely welcomed PITC as an intervention which has saved lives, they lamented the shortage of resources which made it difficult to implement, in many cases forcing them to prioritise pregnant women and patients suspected to be suffering from HIV-related illness. Increased workload, particularly in HIV clinics, was reported to be a problem, as has been reported in other settings [12,30-33]. It was encouraging that many HCW felt that this was made worthwhile by the improved health and decreased workload in the in-patients and out-patients departments. It is clear that with this positive perception PITC will become a huge success once the shortages of resources have been addressed.

**Figure 1:**
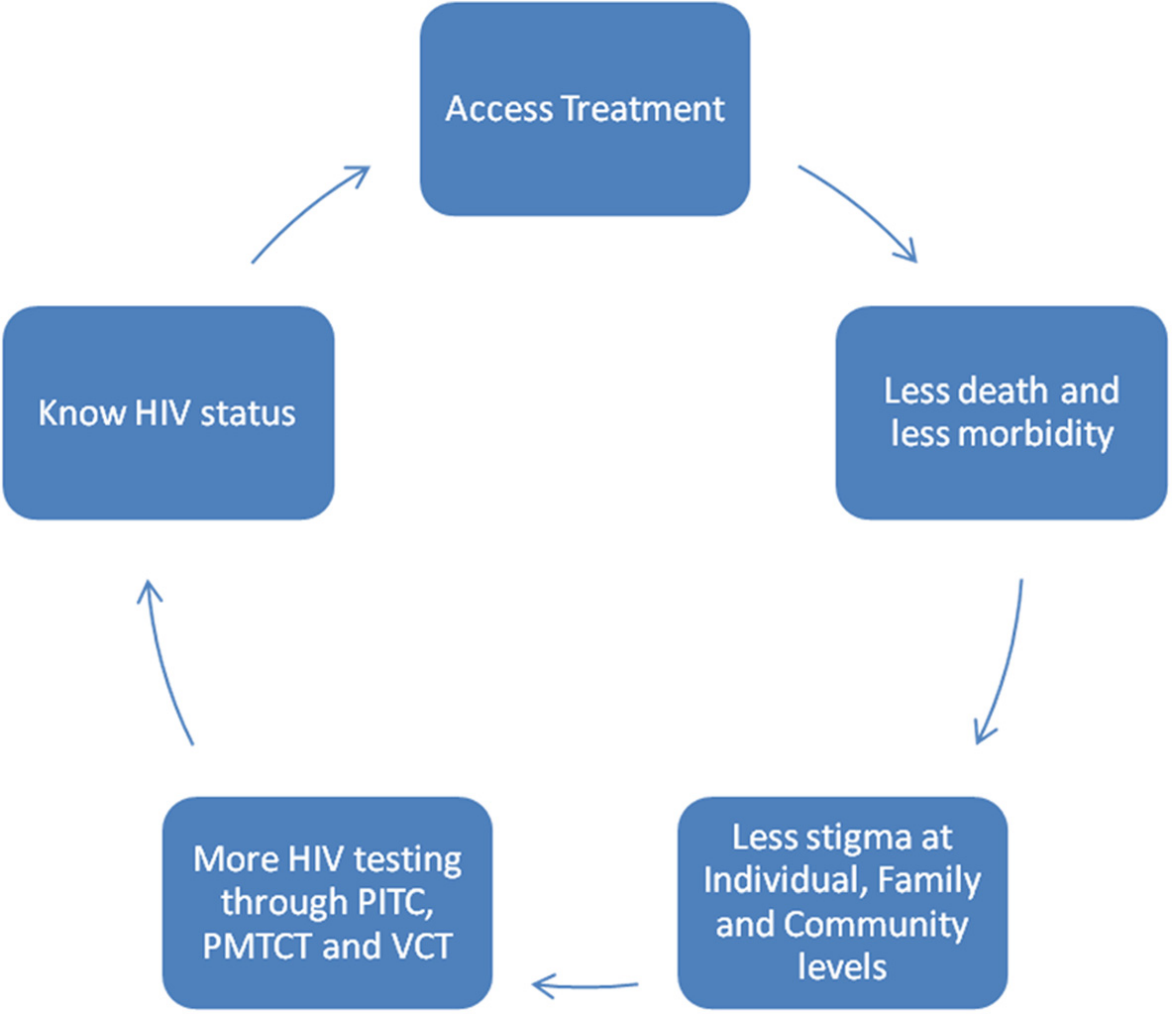
**Benefits of routine HIV testing: A virtuous cycle.** A greater proportion know their HIV status, thus leading to greater access to treatment.

According to the Health Belief Model [34,35], an individual is likely to get tested for HIV if they believe that they are at risk of infection, that HIV/AIDS is a serious disease and they have positive expectations that by getting tested they will avoid severe HIV illness and death. Indeed studies have reported that knowledge of the existence of effective treatment motivates HIV testing [20,22] In this study patients reported feeling motivated to get tested for HIV after realising the benefits of knowing one’s HIV status among friends and family who had been tested. Health care workers reported reduced HIV stigma in communities as a result of more open discussions during PITC. This implies that as HIV infected people get treated for HIV, their health improves and they are less likely to be stigmatised. Studies have reported greater levels of HIV testing among individuals who do not have stigmatising attitudes towards HIV infected persons [20,36]. It is likely that the interplay between perceived benefits of testing and reduced community stigma creates a virtuous cycle whereby HIV testing and early initiation of treatment results in reduced stigma and thereby further increases testing uptake (Figure 1), which is vital in the achievement of WHO goals of universal access to antiretroviral treatment. PITC is therefore an important intervention that ensures better health in countries which face a generalised HIV epidemic.

This study adds to the existing body of evidence of the acceptability of PITC by both patients and health care workers which should serve as an impetus for scale-up of this intervention in countries with generalised HIV epidemics. This study also highlights that for PITC to be optimally provided countries should be prepared to mobilise additional human, material and structural resources, which can be a challenge in resource-limited settings.

The strength of this study is that various methods were employed to collect data on PITC from a variety of sources, which allows validation/triangulation. For example patients were able to confirm whether they had received the services that providers said they provided, and the challenges described by the head of the institution/designee in the semi-structured questionnaire were often echoed by health care workers in the FGDs.

This study had limitations. Firstly, the sites included in this study were specifically selected on the basis that they were submitting data relating to implementation of PITC to MoHCW. Therefore the findings may not be generalisable across Zimbabwe and are likely to represent the best of what was happening at the time of the review. As a result of test kits shortages the study team often had few patients to select for interview. In addition these patients were often not typical of clinic attendees more generally but had a particular reason to be offered testing. They may therefore have felt differently about PITC than other attendees. There were fewer males accessing services than females (which is in keeping with other studies [37]), however the extent of gender imbalance is marked and likely resulted from pregnant women being prioritised for testing at a time of test kit shortages. It is possible that some challenges faced by health care workers might have remained unsaid in the focus group discussions because they were hesitant to criticise the service or their colleagues in front of each other.

### Reflexivity

Qualitative interviews were conducted by experienced field workers with a medical background, mostly nurses. Patients/attendees viewed the field workers as qualified health care personnel who were external to the health care institution environment. This seemed to have enabled them to open up and discuss private thoughts that are usually shared with trusted medical professionals. In addition, patients/attendees viewed the researchers as individuals who could influence the way services were provided at their health care institution through the outcomes of the research. They therefore were keen to share information on what was working well, and what was not. Because the researchers were external to the system, participants were not afraid to register their dissatisfaction as there were no perceived penalties. It is therefore unlikely that patients/attendees gave socially-correct answers.

Health care workers also perceived the research as an approach to solve any problems related to their PITC roles. Rather than give socially desirable responses to portray themselves as doing a good job, they seemed to be open about the challenges they felt were hindering optimum performance of PITC duties.

## Conclusion

PITC has been embraced as a life-saving intervention by both patients and HCW. There is need to address shortages in material, human and structural resources which have prevented optimum implementation.

## Competing interests

No completing interests have been declared by the authors.

## Authors’ contributions

ELS participated in the design of the study, collection of data, conducted data analysis and helped draft the manuscript. KH participated in the design of the study, interpretation of findings and helped draft the manuscript. OM, GN BD, and LM conceived the study and facilitated its design and coordination, helped in the interpretation of findings and commented on the manuscript. PS participated in the design of the study, collection of data, qualitative data analysis and commented on the manuscript. AM participated in the design of the study, helped in the coordination of study activities and commented on the manuscript. GB participated in the design of the study and facilitated its coordination. FMC participated in the design of the study, supervised the research team, was involved in interpretation of study findings and helped draft the manuscript. All authors approved the final submitted version.

## Pre-publication history

The pre-publication history for this paper can be accessed here:

http://www.biomedcentral.com/1472-6963/12/131/prepub
